# PPARgamma dependent PEX11beta counteracts the suppressive role of SIRT1 on neural differentiation of HESCs

**DOI:** 10.1371/journal.pone.0298274

**Published:** 2024-05-16

**Authors:** Maryam Esmaeili, Mohammad Hossein Nasr-Esfahani, Alireza Shoaraye Nejati, Zahra Safaeinejad, Atefeh Atefi, Timothy L. Megraw, Kamran Ghaedi

**Affiliations:** 1 Department of Cellular Biotechnology, Royan Institute for Biotechnology, Cell Science Research Center, ACECR, Isfahan, Iran; 2 Department of Biomedical Sciences, Florida State University College of Medicine, West Call Street, Tallahassee, FL, United States of America; 3 Faculty of Biological Science and Technology, Department of Cell and Molecular Biology and Microbiology, University of Isfahan, Isfahan, Iran; Army Medical University, CHINA

## Abstract

The membrane peroxisomal proteins PEX11, play a crucial role in peroxisome proliferation by regulating elongation, membrane constriction, and fission of pre-existing peroxisomes. In this study, we evaluated the function of *PEX11B* gene in neural differentiation of human embryonic stem cell (hESC) by inducing shRNAi-mediated knockdown of *PEX11B* expression. Our results demonstrate that loss of *PEX11B* expression led to a significant decrease in the expression of peroxisomal-related genes including *ACOX1*, *PMP70*, *PEX1*, and *PEX7*, as well as neural tube-like structures and neuronal markers. Inhibition of *SIRT1* using pharmacological agents counteracted the effects of *PEX11B* knockdown, resulting in a relative increase in *PEX11B* expression and an increase in differentiated neural tube-like structures. However, the neuroprotective effects of *SIRT1* were eliminated by PPAR inhibition, indicating that *PPARɣ* may mediate the interaction between *PEX11B* and *SIRT1*. Our findings suggest that both *SIRT1* and *PPARɣ* have neuroprotective effects, and also this study provides the first indication for a potential interaction between *PEX11B*, *SIRT1*, and *PPARɣ* during hESC neural differentiation.

## Introduction

Peroxisomes are a versatile class of ubiquitous membrane-bound organelles that respond dynamically to metabolic, developmental, and environmental cues by altering their morphology, abundance, enzyme content, and shape in eukaryotic organisms [1–5]. In mammals, peroxisomes harbor approximately 50 matrix enzymes involved in a diverse range of metabolic reactions, including very long chain fatty acid degradation, plasmalogen biosynthesis, glycolysis, and reactive oxygen species elimination [2,5,6]. Peroxisome biogenesis requires the coordinated action of over 30 peroxins, a group of peroxisomal proteins, among which the PEX11 family has been shown to play a direct role in peroxisome proliferation in yeast, plants, and mammals [1,3,7]. Mutations in *PEX11B* have been associated with peroxisome biogenesis disorders (PBDs) [1], and studies on animal models lacking either *PEX11A* or *PEX11B* have provided significant insights into peroxisome biology [3]. The spectrum of PBDs, such as Zellweger syndrome, highlights the critical role of peroxisomes in human health [8,9]. In mouse brain tissue, *Pex11b* deletion results in oxidative stress and neural phenotypes, although the severity is less pronounced than in homozygous animals [10]. Manipulation of *PEX11B* expression levels, either through overexpression or knock-down, alters the expression of peroxisomal-related genes and thus impacts peroxisome proliferation [11].

Sirtuins (SIRTs) are deacetylases that belong to the class III histone deacetylase family [12]). These enzymes, including seven isoforms (SIRT1 to SIRT7), vary in subcellular localization, substrate specificity, and response to stimuli [13]. *SIRT1*, the most well-characterized isoform, is expressed in the nucleus and regulates a variety of biological processes, such as gene expression control, oxidative stress, genome stability, homeostasis, aging, cellular proliferation, and metabolism [14], modulation of synaptic plasticity and memory formation in the adult brain [15]. Sirtuins, particularly *SIRT1*, possess neuroprotective characteristics [16] by deacetylating various substrates including tumor suppressor P53 [17], peroxisome proliferator-activated receptor gamma (PPARɣ), PPARɣ coactivator-1α (PGC1α) [18], nuclear factor kappa β (NF-κβ), and liver X receptor (LXR) [19].

*PPARɣ*, one of three significant isoforms in the nuclear receptor superfamily [20], is present in high levels in brain tissue [21,22] and with lower expression in heart, liver, and skeletal muscle [21]. *PPARɣ* activates transcription of target genes by binding to specific DNA regions called peroxisome proliferation hormone response elements (PPREs). Through transcriptional activation, *PPARɣ* regulates adipogenesis, energy balance, lipid biosynthesis [23], and neuroprotective mechanisms in neurophysiology [21].

In this study, we aimed to investigate the relationship between *SIRT1*, *PEX11B*, and *PPARɣ*, considering the established neuroprotective roles of *PPARɣ* [24,25], the ability of pioglitazone, as a *PPARɣ* agonist, to alleviate the effects of *PEX11B* knockdown [26], and the proposed direct substrate role of *PPARɣ* for *SIRT1* as well as indirect effect of *PPARɣ* on *SIRT1* via *SIRT1-PGC1α* axis [18,35]. Previously, we demonstrated that *PEX11B* knockdown reduced the expression of neuronal markers and peroxisomal-related genes during neural differentiation of mouse embryonic stem cell (mESCs) [26]. Here, we replicated and expanded on these findings by performing experiments using hESC culture and neural differentiation. To explore the interplay between *PEX11B*, *SIRT1*, and *PPARɣ*, we employed a SIRT1 inhibitor (EX-527) and observed that SIRT1 inhibition alleviated the blockade of neural differentiation caused by *PEX11B* knockdown. Moreover, the inhibitor of *PPARɣ* (GW9662) reversed the effect of SIRT1 inhibition, confirming that the influence of SIRT1 inhibition on neural tube formation was mediated by *PPARɣ*.

## Materials and methods

### Cell culture and neural differentiation

The human embryonic stem cell line RH6 [25] was cultured on Matrigel (Sigma-Aldrich, E127) and maintained in specific hESC medium consisting of Dulbecco’s modified Eagle medium /Ham’s-Nutrient Mixture F-12 (DMEM/F12, Gibco, 21331–020), supplemented with 20% knock-out serum (KSR, Gibco, 10828–028), 100 units/mL penicillin, 100 ng/mL bFGF (Royan Institute), 2 mM L-glutamine (Gibco, 25030–024), 1% insulin-transferrin-selenite (ITS, Gibco, 41400–045),100 μg/mL streptomycin (Gibco, 15070063),1% nonessential amino acids (Gibco, 11140–035), and 0.1 mM β-mercaptoethanol (Sigma-Aldrich, M7522). Cells were maintained at 37°C with 5% CO2 and passaged using accutase (Millipore, SCR005). Neural differentiation was performed using a previously described protocol [26] with some modifications. The culture medium was changed every other day after day 4, and neural tube-like structures were manually selected on day 14 and cultured in adherent Matrigel-coated dishes. After 7 days, neurite outgrowth was observed.

### Vector construction

We employed lentiviral vectors, including pLVTHM and pLVPT-tTR-KRAB (Addgene), to introduce short hairpin RNA (shRNA) targeting *PEX11B* into the genome of hESCs, following the supplier’s protocol (Addgene, Cambridge, MA, USA; Table 1). Initially, the shRNA sequence directed towards *PEX11B* was integrated downstream of the tetO-H1 region in pLVTHM vector. Subsequently, the cassette was excised from pLVTHM containing shRNA and sub-cloned into pLVPT-tTR-KRAB, an expression vector that regulates transcription in a doxycycline-dependent manner. As such, two vectors were generated: pLVPT-tTRKRAB/ShPEX11B and pLVPT-tTR-KRAB/ShCtrl.

**Table 1:** Designed *PEX11β*-targeted shRNAs and shCtrl sequences.

Name		*Mlu*I site	Sense	Hairpin loop	Antisense	Terminator	BSTZ171, ClaI
shRNA	**U**	5´-**CGCGT**CCCC	GCTGTTCACCTATCAGATGTTGTCCTGAG	TTCAAGAGA	CTCAGGACAACATCTGATAGGTGAACAGC	TTTTT	GGAA**GTATACAT**-3´
	**L**	3´-**A**GGGG	CGACAAGTGGATAGTCTACAACAGGACTC	AAGTTCTCT	GAGTCCTGTTGTAGACTATCCACTTGTCG	AAAAA	CCTT**CATATGTAGC**-5´
shCtrl	**U**	5´-**CGCGT**CCCC	GACCATCAATATGACTAGA	TTCAAGAGA	TCTAGTCATATTGATGGTC	TTTTT	GGAA**GTATACAT**-3´
	**L**	3´-**A**GGGG	CTGGTAGTTATACTGATCT	AAGTTCTCT	AGATCAGTATAACTACCAG	AAAAA	CCTT**CATATGTAGC**-5´

U: Upper oligonucleotide strand. L: Lower oligonucleotide strand. The partial sequences of the *Mlu*I restriction site are underlined upstream of upper strand of oligonucleotides and downstream of lower oligonucleotide strands. Additionally, the partial sequences of *Cla*I restriction site and complete sequences of BSTZ171 restriction site are underlined downstream of upper strand of oligonucleotides and upstream of lower oligonucleotide strands.

### Generation of stable cell line using lentiviruses

Lentiviral particles were generated by transiently transfecting HEK293T cells with 2nd generation packaging vectors (PsPAX2 and pMD2G) along with the inducible vector pLVPT-tTR-KRAB containing shPEX11B or shCtrl. The transfection was carried out using lipofectamine LTX (Invitrogen) as previously described. After 72 h, the supernatant was collected, filtered, and concentrated. The titration of the lentiviral particles was carried out via FACS analysis (Becton Dickinson, Franklin Lakes, NJ, USA) according to a previously reported method [27]. Next, hESCs were seeded into 6-well plates and transduced with freshly prepared lentiviral particles at a multiplicity of infection (MOI) level of 10. Two days after transduction, the cells were treated with blasticidin (6 μg/mL) (Gibco, Grand Island, NY, USA) for two weeks until stable colonies appeared.

### Dox, EX-527 and GW9662 treatments

To induce the expression level of shPEX11**B** and shCtrl 750 ng/mL doxycycline (Dox) we used (Dox, Clontech) during neural differentiation of hESCs. Following Dox treatment, the inhibitory effects of tTR-KRAB on nearby promotor activity was eliminated and shRNA was expressed [28]. EX-527 (6-chloro-2, 3, 4, 9-tetrahydro-1H-carbazole-1- carboxamide; Sigma-Aldrich, E7034) and GW9662 (Sigma-Aldrich, M6191) were dissolved in dimethyl sulfoxide (DMSO) with final concentrations of 5 mM and 10μM respectively. In all experiments, equal amount of solvent (vehicle) was used for controls.

### RNA isolation and RT-qPCR analysis

Total RNA was isolated using the RNeasy Kit (Qiagen, 74004) and treated with DNaseI (Thermo Scientific, EN0521) to synthesize cDNA from 1 μg of total RNA using cDNA synthesis Kit (Thermo Fisher Scientific, K1622). real-time quantitative PCR (RT-qPCR) was performed using SYBR green Gene Expression Master Mix (TaKaRa, RR820Q) and 25 ng of cDNA on a Rotor-Gene 6000 thermal cycler (Corbett). The expression levels of all target genes were normalized to that of glyceraldehyde 3-phosphate dehydrogenase (GAPDH) as a reference gene in three independent experiments. The data were analyzed using the ΔΔCt method [29]. The primer pairs for each gene were designed using Beacon Designer software (Version 7.2, USA) and synthesized by Metabion Company (Germany). The primer sequences for each gene are provided in Table 2.

**Table 2:** List of primers used in this study.

Target gene		Primer sequence (5′ to 3′)
***ACOX1***	F	TCTTGTTGATGTTCTGACTT
	R	GTGTGACTTGTTCTAATCCT
***TUJ1***	F	AAGCCAGCAGTGTCTAAACCC
	R	GGGAGGACGAGGCCATAAATAC
***GAPDH***	F	CCACTCCTCCACCTTTGACG
	R	CCACCACCCTGTTGCTGTAG
***NANOG***	F	CAGCTACAAACAGGTGAAGAC
	R	TGGTGGTAGGAAGAGTAAAGG
***OCT4***	F	TCTATTTGGGAAGGTATTCAGC
	R	ATTGTTGTCAGCTTCCTCCA
***PAX6***	F	TTGCTGGAGGATGATGAC
	R	CTATGCTGATTGGTGATGG
***PEX3***	F	GATGCTGAGGTCTGTATGG
	R	CCCTTTCCTGTATTTCTCTGAT
***PEX7***	F	GCCTCTTGCTCGTATGATT
	R	CTGTATGATGCTCCACTGTT
***PEX1***	F	TCAGTTGGATGGAGTAGAAG
	R	TCAGGAGGAGGACAGTAT
***PEX11β***	F	CAGAGGTGGCTTATGGCAGAT
	R	GCGAGGTTGGTGAGGTAGAC
***PEX13***	F	AGTGCGTGGTTGGCTTCTG
	R	CCGTGGCTCCTTTAGTTAGTGTTG
***PMP70***	F	GGAAAACCACCATTACAGAACA
	R	CGAGACACCAGCATAACAG
***SOX1***	F	GCATTTCTTTCCTGTGGTTCTG
	R	TGGCTGTTGTCCCTATCCT

### Western blot analysis

Three separate cell cultures of hESCs were washed with PBS and directly lysed in TRIzol reagent (Sigma-Aldrich, St. Louis, MO, USA, catalog no: 93289) according to the manufacturer’s instructions. The protein content of the lysed cells was estimated using the Bradford method. Equal amounts of protein (30 μg) from each lysate were separated by SDS-12% polyacrylamide gel electrophoresis and transferred onto a polyvinylidene difluoride (PVDF) membrane (Bio-Rad, 162–0176). The primary antibodies were PEX11b (Assay Biotech, C17630, dilution 1:1000), SIRT1 (Abcam, AB110304, dilution 1:4000), and glyceraldehyde 3-phosphate dehydrogenase (GAPDH, Sigma-Aldrich, A2228, dilution 1:5000). The secondary antibody was HRP-conjugated goat anti-mouse IgG (Dako, P0447, dilution 1:5000). Protein bands were visualized using an Amersham ECL Advance Western Blotting Detection Kit (GE Healthcare, Buckinghamshire, UK). Finally, the intensity of each band was quantified using ImageJ software, and the results were normalized against the GAPDH band.

### Statistical analysis

The data of different groups were presented as mean±SEM (standard error of mean). The difference between groups was analyzed using the one-way analysis of variance (ANOVA) and student *t*-test. Differences were considered to be significant at *P* <0.05.

## Results

### Establishment of transduced stable hESCs lines using lentiviral vectors

**Fig 1:**
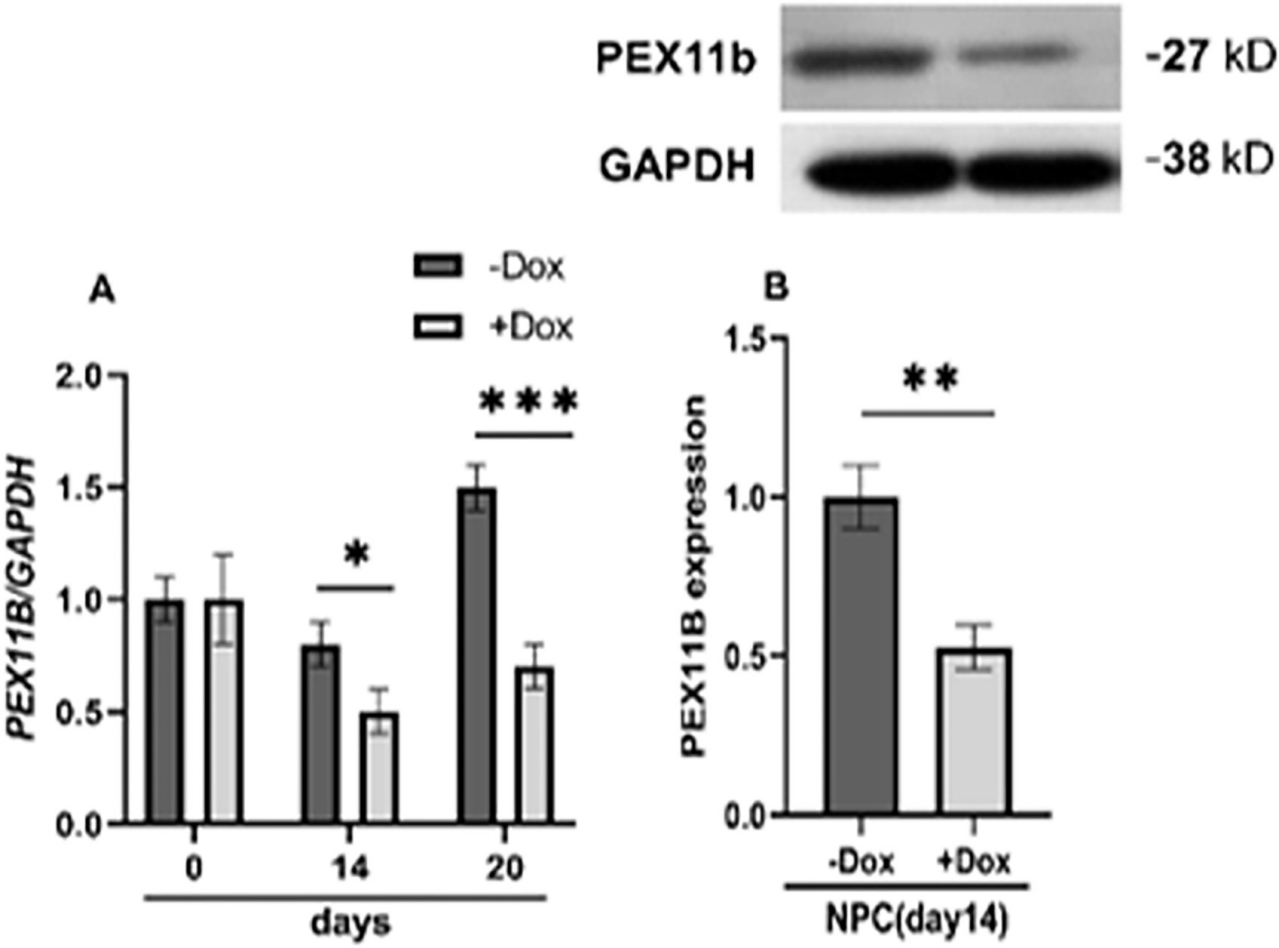
Establishment of stable Dox-inducible *PEX11B* shRNA hESC cell lines (shPEX11B). A) During neural differentiation of transduced stable hESCs lines, the RNA level of PEX11B decreased significantly after Dox treatment. B) Relative protein expression was quantified and normalized with *GAPDH*. Displayed value bars are the mean of duplicate independent experiments± SEM. * indicates a significant difference at *P* <0.05.

hESCs were transduced with a lentiviral construct expressing shRNA against *PEX11B* (pLVPT-tTR-KRAB) and a control vector as previously described. Lentiviral particles, obtained from the supernatant fraction of HEK293T cell culture, were used for transduction of hESCs, with transfection and transduction efficiencies estimated at 99.8% and 60.1%, respectively, by flow cytometry (data not shown). Positive colonies were selected after treatment with blasticidin (6 μg/mL) and were confirmed by genomic insert check PCR before being treated with doxycycline (750 ng/mL) for two days. The stemness characteristics of stable cell lines were evaluated based on the expression levels of *NANOG* and *OCT4*, and no significant changes were observed (S1 Fig). The induced expression of shRNA with doxycycline resulted in a significant reduction in *PEX11B* expression compared to the control shRNA, and was further confirmed by a decrease in PEX11B protein levels via Western blot analysis (Fig 1).

### Knock-down of *PEX11B* reduces expression of neural tube and neuronal markers and peroxisomal-related genes

**Fig 2:**
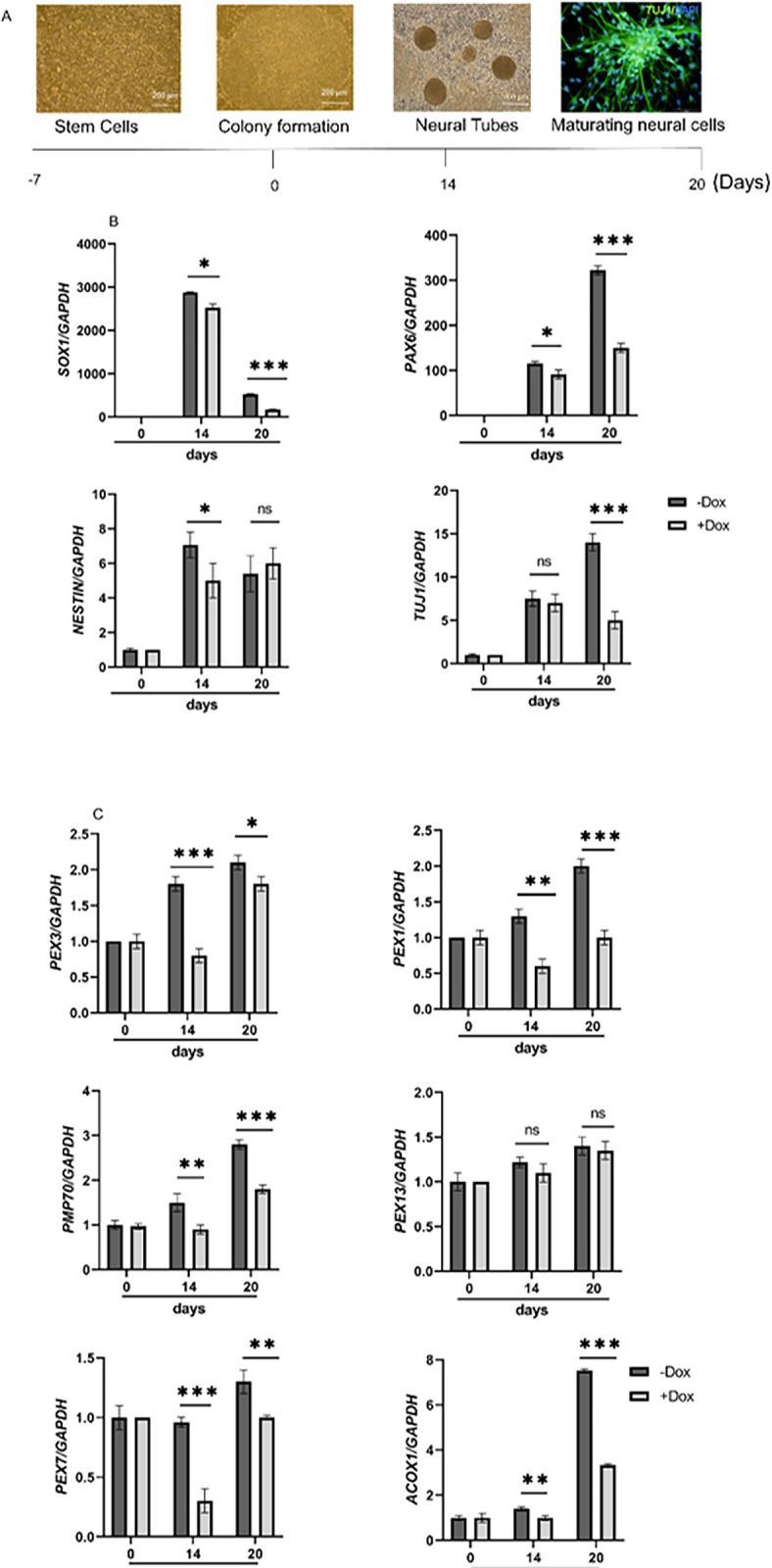
mRNA level of neural progenitor cells and neuronal markers and peroxisomal-related genes were changed after treatment with Dox during neural differentiation of stable hESCs lines. A) schematic diagram of neural differentiation and Dox treatment (day 0). B) RT-qPCR for evaluation of *SOX1*, *PAX6*, *NESTIN* and *TUJ1* RNA level before and after Dox treatment. C) RT-qPCR for estimation peroxisomal-related genes *PEX1*, *PEX3*, *PMP70*, *PEX13*, *PEX7* and *ACOX1* before and after Dox treatment. Relative expression was quantified and normalized with *GAPDH*. Displayed value bars are the mean of triplicate independent experiments± SEM. * represents significant difference at *P* <0.05.

Stable lines of hESCs containing *PEX11B* and control shRNAs were differentiated into neural cells, as shown in Fig 2A, through serum reduction. The relative expression levels of neural progenitor markers *SOX1* and *PAX6*, as well as the neuronal marker *TUJ1*, were reduced upon knock-down of *PEX11B*, as illustrated in Fig 2B. The reduction in *SOX1* and *NESTIN* expression levels was observed only on day 14, during neural tube formation, while *PAX6* expression continued to decrease throughout the differentiation period (day 20). Additionally, transcript levels of peroxisomal-related genes were analyzed pre- and post-Dox treatment. Dox-induced knock-down of *PEX11B* significantly decreased the expression levels of *PEX1*, *PEX3*, *PMP70*, *PEX7*, and *ACOX1*, but not *PEX13*, as presented in Fig 2C. No changes in the expression profiles of these genes were observed in the control or untransfected lines.

### *SIRT1* inhibition enhances expression of markers of neural tube-like structures and peroxisomal-related genes in hESCs lines with *PEX11B* knocked-down

**Fig 3:**
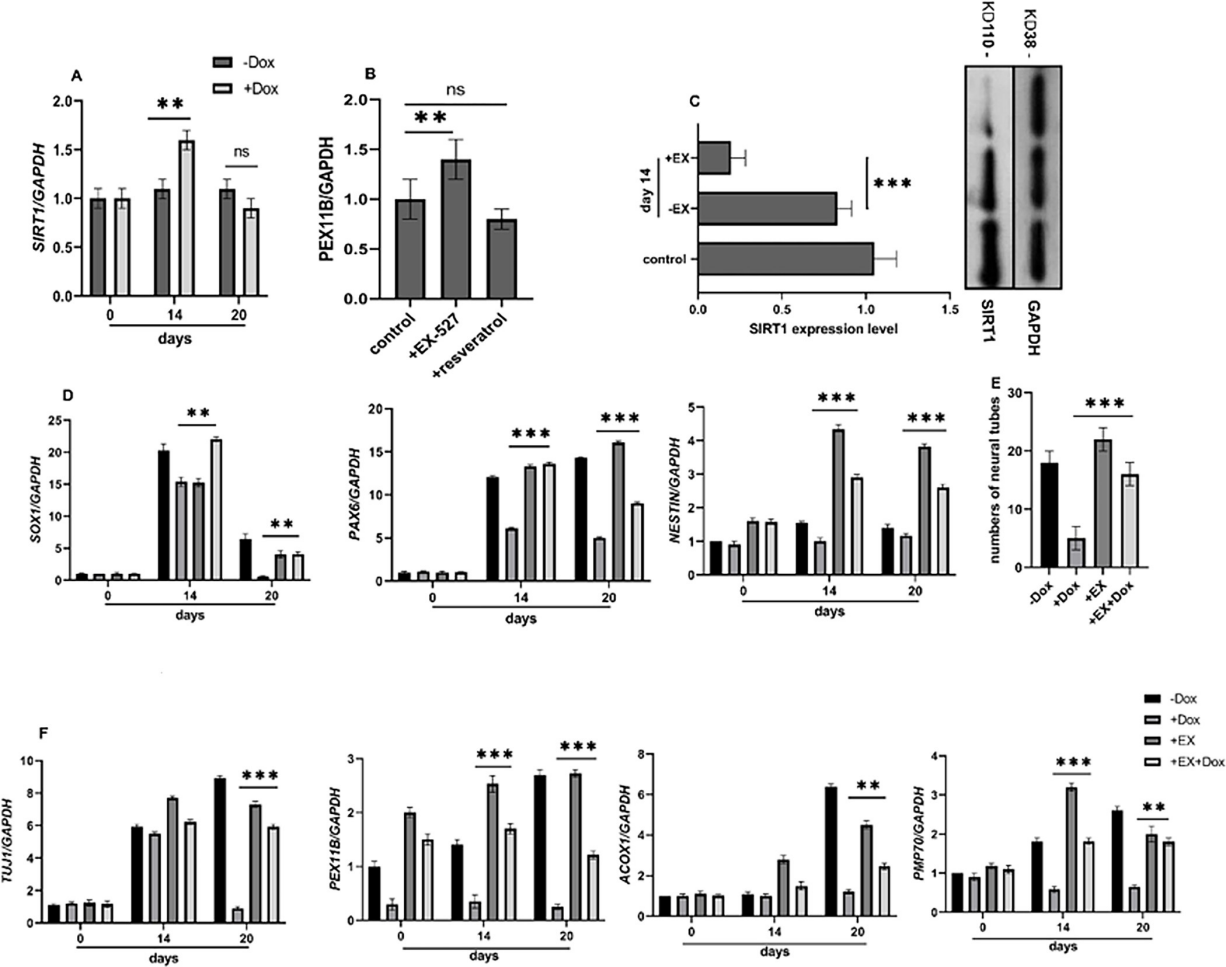
EX-527 treatment of stable hESCs lines affects SIRT1, peroxins, and neuronal marker expression during neural differentiation. A) relative mRNA expression of *SIRT1* before and after Dox treatment during neural differentiation. In neural precursor cells the expression level of SIRT1 was increased after Dox treatment. B) and C) mRNA and protein level of *SIRT1* was decreased after Ex-257 treatment. D) relative expression of *SOX1*, *PAX6*, *NESTIN* and *TUJ1* were increased after EX-527 and Dox treatment compared to Dox treatment alone. E) number of neural tube-like structure was increased after Ex-257 treatment. F) RT-qPCR for evaluation of peroxisomal-related genes *PEX11B*, *ACOX1* and *PMP70*. Relative expression was quantified and normalized with GAPDH. Displayed value bars are the mean of triplicate independent experiments± SEM. * represents significant difference at *P* <0.05.

Considering the increased expression of *SIRT1* during the time course of in vitro neural development of hESCs lines in the presence of Dox (Fig 3A), to explore the potential association between *SIRT1* and *PEX11B* (Fig 3B and 3C), we used EX-527 and resveratrol to activate and inhibit *SIRT1*, respectively. Intriguingly, inhibiting *SIRT1* with EX-527 and activating it with resveratrol caused an increase and decrease in the expression of PEX11B, respectively. When Dox-induced shPEX11B lines were treated with EX-527, the relative expression of markers for neural tube-like structures, including *SOX1*, *PAX6*, *NESTIN*, and the neuronal marker *TUJ1* were increased (Fig 3D). Moreover, the number of neural tube-like structures also increased in Dox-induced shPEX11B cell lines treated with EX-527 (Fig 3E). Additionally, the mRNA levels of the peroxisomal-related genes *ACOX1* and *PMP70* were significantly increased after EX-527 treatment in shPEX11B cell lines during neural differentiation. These findings collectively suggest that *SIRT1* has a negative regulatory effect on peroxisomal biogenesis genes during neural development in shPEX11B lines.

### *PPARɣ* mediates the interaction between *SIRT1* and *PEX11B*

**Fig 4:**
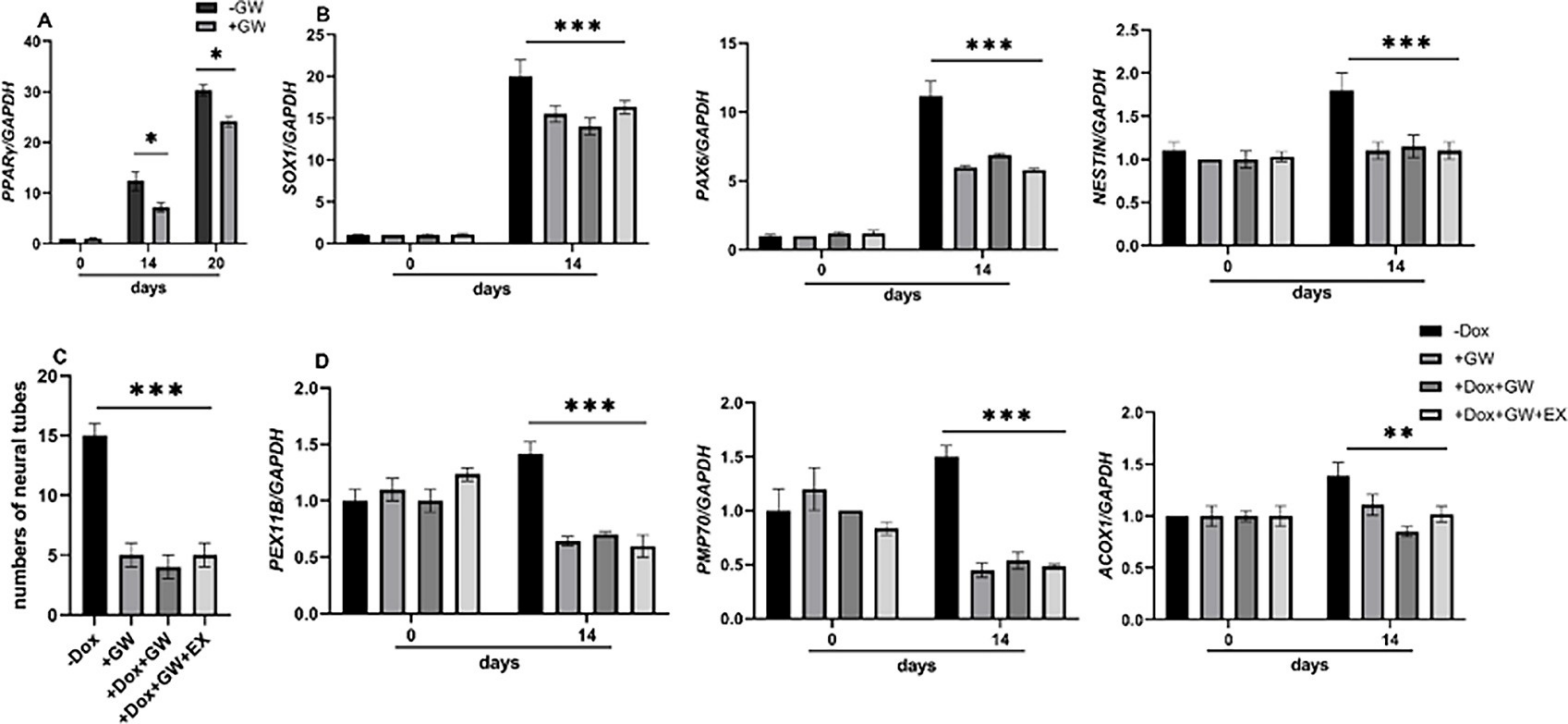
RT-qPCR analysis for neuronal markers and peroxisomal-related genes during neural differentiation of neural progenitor cells before and after GW9662 treatment (GW). A) relative expression of *PPARɣ* before and after GW9662 treatment. B), C) and D) relative expression of neural progenitor cells and neuronal markers, number of neural tubes and peroxisomal-related genes during neural differentiation were decreased after GW treatment and EX-527 (EX) did not ameliorate the effects of *PEX11B* knock-down (Dox).

**Fig 5:**
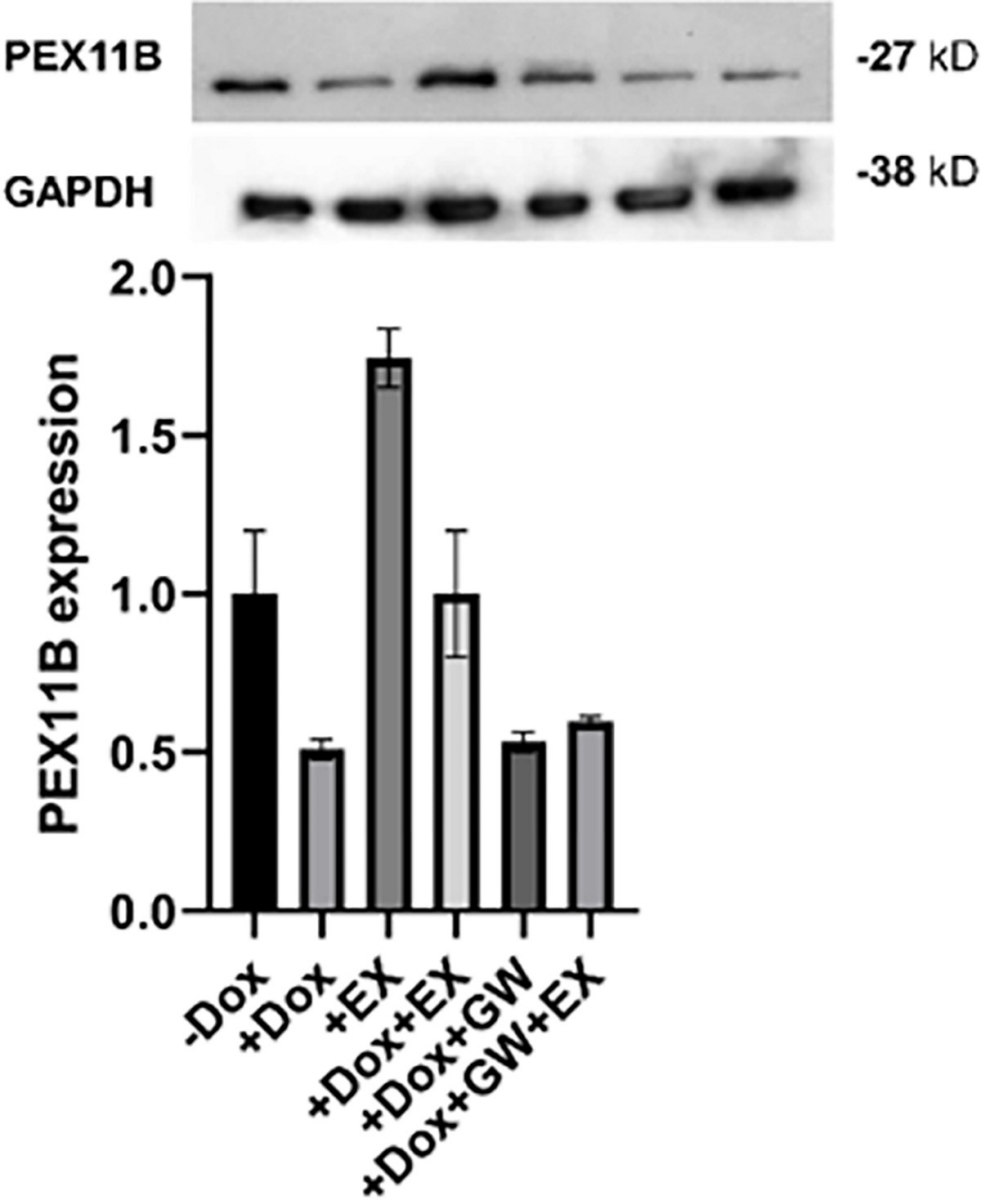
Western blot analysis of PEX11B in different modes of treatment with DOX, EX-527 and GW9662.

To investigate whether *PPARɣ* mediates the relationship between *SIRT1* and *PEX11B*, we employed GW9662, a *PPARɣ* antagonist, during neural differentiation of Dox-induced shPEX11B cell lines. Surprisingly, we observed no changes in the expression levels of neural tube-like markers and peroxisomal genes upon treatment with EX-527 after knockdown of *PEX11B* (Fig 4), suggesting that *PPARɣ* may not play a mediating role in this relationship. The western blot analysis of PEX11B (Fig 5) also validated the RT-qPCR results presented in Fig 4.

## Discussion

The conserved regulatory function of the PEX11 protein family has been widely identified among the genes and proteins involved in peroxisome biogenesis [30]. *PEX11B*, in particular, has been identified as a key regulator of peroxisome elongation and division, as overexpression of *PEX11B* alone can induce these processes [31]. In contrast, a reduction in *PEX11B* gene expression has been shown to lead to a decrease in peroxisomal abundance [32]. Given the pivotal role of *PEX11B* in peroxisome biogenesis, we investigated the effects of *PEX11B* knockdown on neural differentiation of hESCs. Our findings demonstrate that knockdown of *PEX11B* expression during neural differentiation significantly reduces the expression of neural tube-like structure and mature neuronal markers, as well as the mRNA levels of peroxisomal-related genes such as *PMP70*, *ACOX1*, *PEX1*, *PEX3*, and *PEX7* in neural tube-like and neuronal cells. However, the relative expression of PEX13 was not significantly change during neural differentiation. Taken together, our data, in conjunction with our previous study [26], suggest that neural precursor cells are particularly sensitive to the loss of *PEX11B*, resulting in a reduction in the size of embryoid bodies (EBs) from mESCs and a decline in the number of neural tube-like structures from hESCs, ultimately leading to a reduction in neurogenesis during in vitro neural differentiation. In mouse embryonic fibroblasts and undifferentiated muscle cells, SIRT1 has been reported to control the proliferation, cell cycle arrest, and differentiation as a redox sensor [33]. Furthermore, *SIRT1* expression was shown to decrease during the differentiation and maturation of embryonic cortical neurons [34,35]. Building on these findings, we evaluated the expression levels of *SIRT1* during neural differentiation of hESCs and shPEX11B lines. Our results revealed that the mRNA levels of *SIRT1* in neural tube-like structures increased after *PEX11B* knockdown. We then investigated the effects of increased expression levels of *SIRT1* in neural progenitors by using EX-527 as a SIRT1 inhibitor. The expression levels of neural tube and neural markers, as well as peroxisomal-related genes, were increased following *SIRT1* inhibition with EX-527 treatment. Moreover, the abundance of neural tube-like structures was significantly increased. We propose that inhibition of *SIRT1* improves neural differentiation by increasing the expression levels of *PEX11B*, which enhances peroxisomal biogenesis through the expression of peroxisomal-related genes. consequently, we hypothesized that *PPARɣ*, which activates the transcription of *PEX11B* [26] while is also considered as an inhibitory substrate of *SIRT1* [18], may serve as the interface mediator between *PEX11B* and *SIRT1*. To test this hypothesis, we used GW9662 as an antagonist of *PPARɣ*. Interestingly, the effect of *SIRT1* inhibition on elevating *PEX11B* expression level was eliminated by treatment with GW9662.

**Fig 6:**
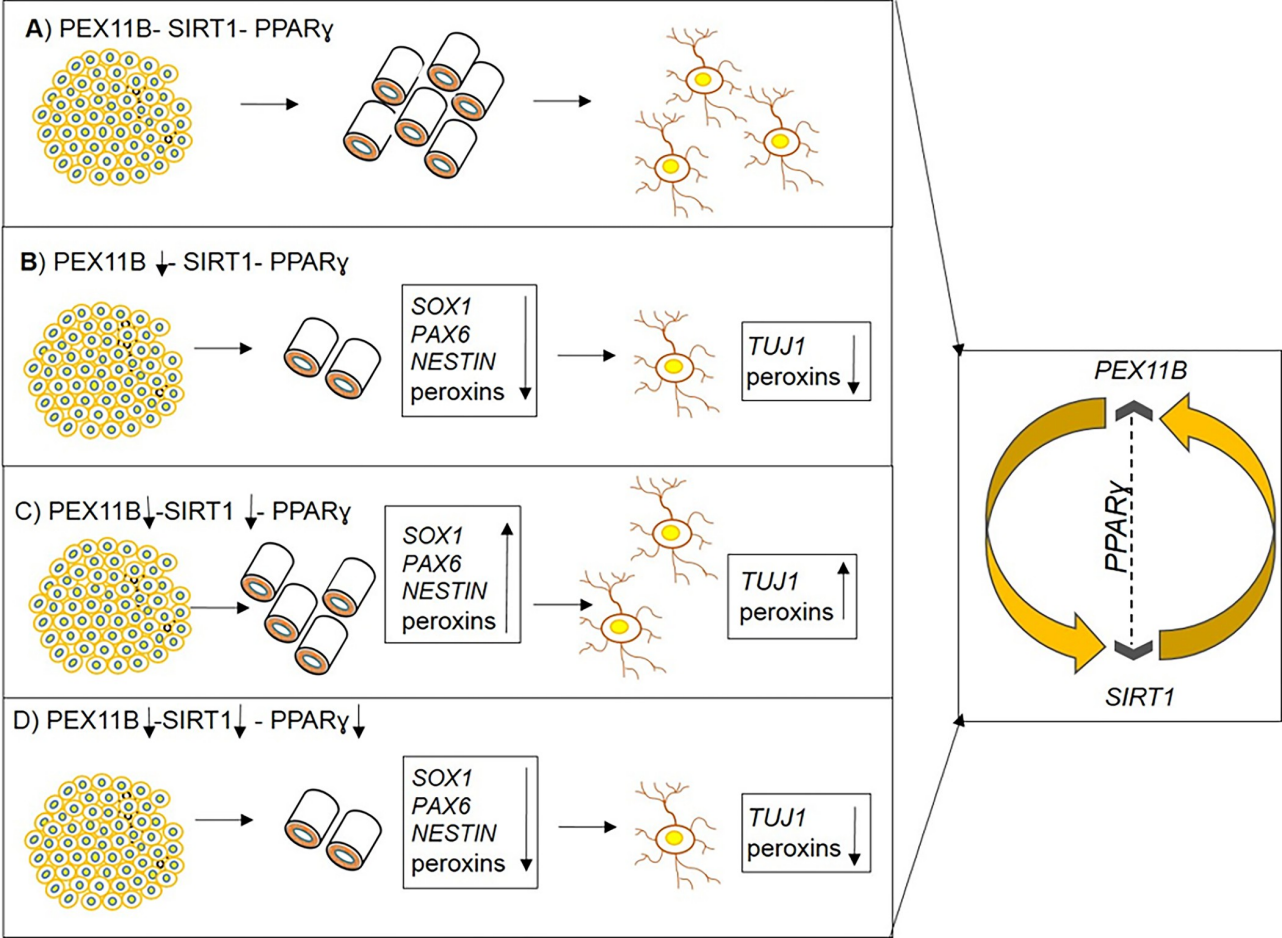
Regulation of neural differentiation at least partly via *PEX11B-SIRT1-PPARɣ* axis. A) normal neural differentiation. B) *PEX11B* knock-down caused to decrease of number of neural tubes and neurogenesis. C) amelioration of damages caused by *PEX11B* knock-down through inhibition of *SIRT1*. D) inhibition of *PPARɣ* reverses the ameliorative effects of inhibition of *SIRT1*. Accordingly, it seems that the interplay between *PEX11B* and *SIRT1* is mediated by *PPARɣ*.

While GW9662 itself was found to reduce biomarkers expression of neural precursor and neuron cells, no additional effect was observed when it was combined with Dox. Our results suggest that the inhibition of *SIRT1* may partially compensate for the effects of *PEX11B* knockdown, and that *PPARɣ* is likely the mediator between *PEX11B* and *SIRT1*. These findings confirm the neuroprotective effects of *SIRT1* and *PPARɣ* previously reported in the literature, and this is the first report that demonstrates a potential interaction between *PEX11B* and *SIRT1*, which warrants further investigation such as immunofluorescence localization detection of PEX11B-SIRT1-PPARɣ axis (Fig 6).

## Supporting information

S1 Fig mRNA level of *NANOG* and *OCT4* before and after transfection. Original western blot figures related to Fig 1, Fig 3 and Fig 5.
(DOCX)

S1 Raw images (PDF)

## Abbreviations

hESC: human embryonic stem cell
PBDs: peroxisomal biogenesis disorders
SIRT: sirtuins
PPARγ: PGC1α, PPARɣ coactivator-1α;Peroxisome proliferator-activated receptor γ
NF-κβ: nuclear factor kappa β
LXR: liver X receptor
PPRE: peroxisomal proliferator response element
mESC: mouse embryonic stem cell
DMEM/F12: Dulbecco’s modified Eagle medium /Ham’s-Nutrient Mixture F-12
KSR
ITS: insulin-transferrin-selenite
shRNA: Short hairpin RNA
shCtrl: Control shRN
MOI: multiplicity of infection
Dox: Doxycycline
DMSO: dimethyl-sulfoxide
RT-qPCR: real-time quantitative PCR
GAPDH: glyceraldehyde 3-phosphate dehydrogenase
PVDF: olyvinylidenedifluoride
HRP: horseradish peroxidase
ANOVA: one-way analysis of variance
Acox1: acyl-coenzyme A oxidase 1
NPCs: Neural precursor cells
Pax6: paired box gene 6
PBS: phosphate-buffered saline
PMPs: peroxisomal membrane proteins
SEM: standard error of mean
Sox1: SRY-box containing gene 1
VLCFA: very long chain fatty acid
