# Supplementary figures and images for: PPARgamma dependent PEX11beta counteracts the suppressive role of SIRT1 on neural differentiation of HESCs
Source: PLoS One. 2024 May 16;19(5):e0298274. doi: 10.1371/journal.pone.0298274 (PMC11098471; doi:10.1371/journal.pone.0298274)

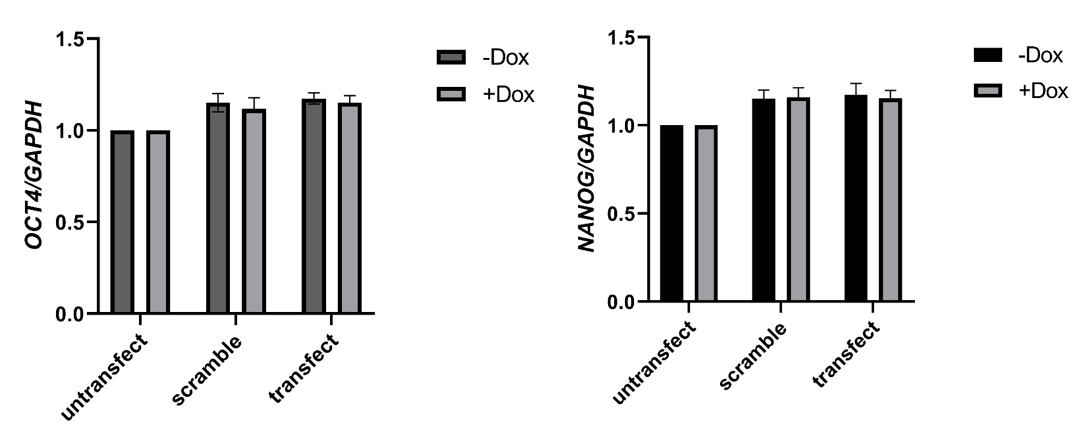

Supplement: S1 Fig — Original western blot figures related to Fig 1, Fig 3 and Fig 5. (DOCX) [file pone.0298274.s001.docx]
