# Supplementary material for: PPARgamma dependent PEX11beta counteracts the suppressive role of SIRT1 on neural differentiation of HESCs
Source: PLoS One. 2024 May 16;19(5):e0298274. doi: 10.1371/journal.pone.0298274 (PMC11098471; doi:10.1371/journal.pone.0298274)
Supplement: S1 Raw images — (PDF) [file pone.0298274.s002.pdf]

Original western blot

related to figure 1

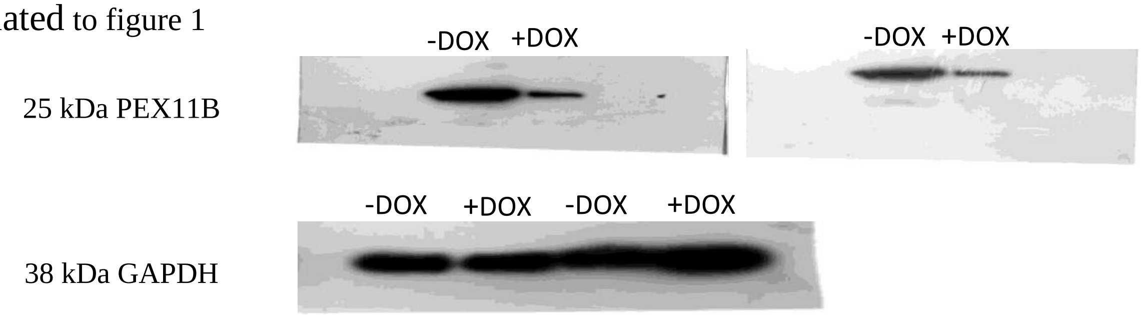


related to figure 3

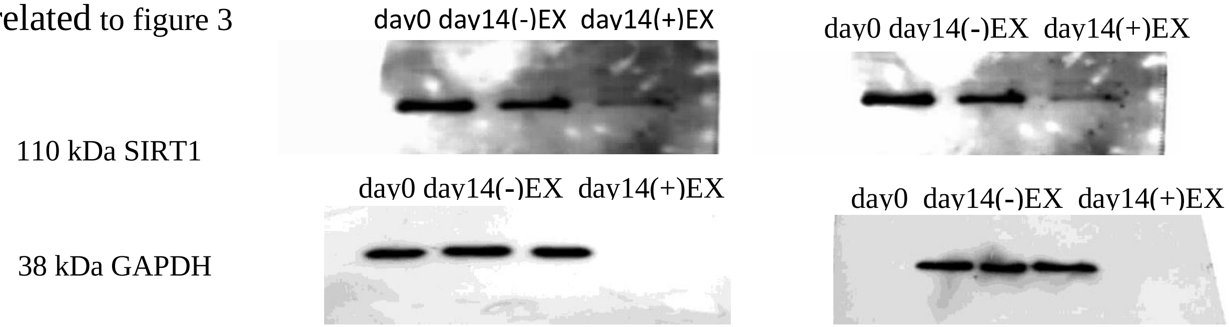


related to figure 5

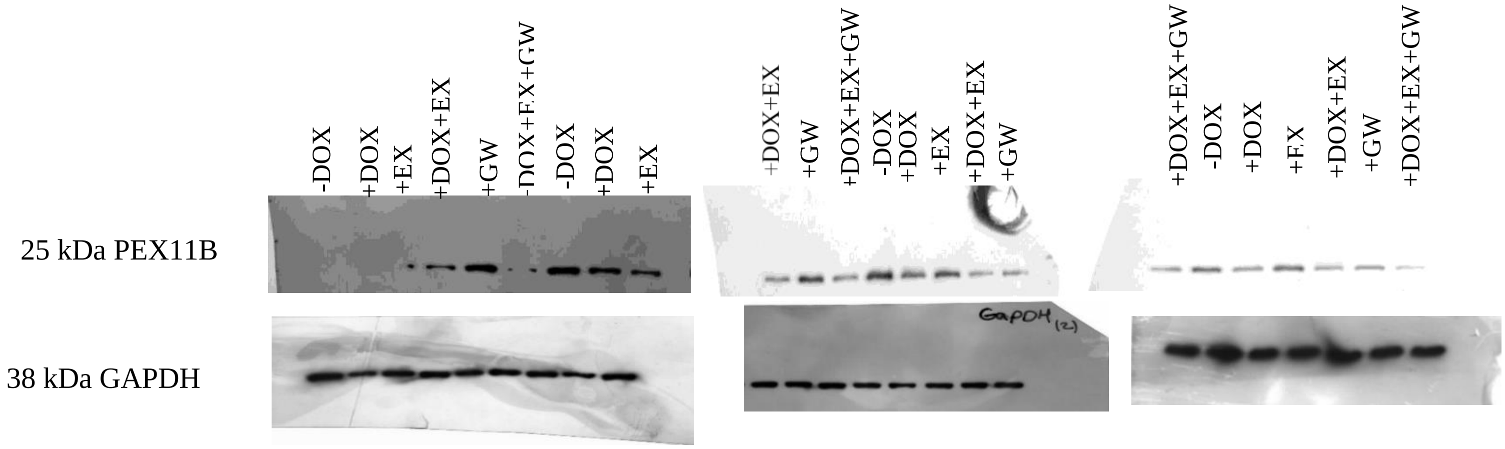

Please note that:

- Figure shows the whole blot after cutting membrane at molecular weight 25 kDa for PEX11B, 38 kDa for GAPDH and 110 kDa for SIRT1.

- The ladder used in this research is the Thermo Scientific™ PageRuler™ Prestained Protein. It can be seen on the original gel, and knowing the approximate size of the desired band, we are able to recognize it, but because this is not a HRP-conjugated ladder, it cannot be seen after the appearance of the western image. It should be noted that due to the high price of conjugated form of this product, we are not able to buy it.

- To reduce costs, PVDF membranes were cut into two or three pieces before exposure to the first antibody or the specific antibody. The film is also cut to fit the size of the membrane.

S1 Fig

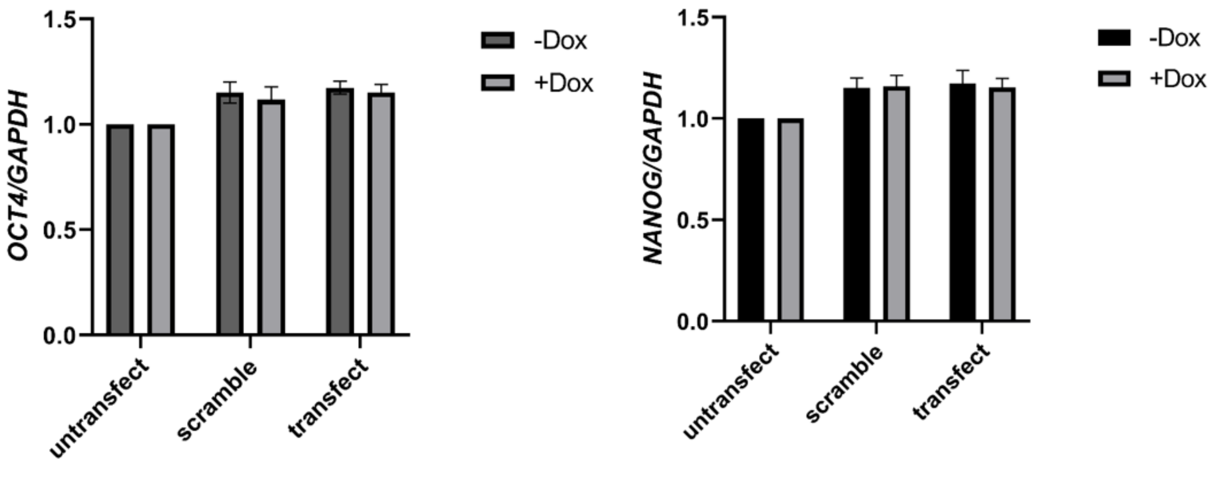


S1 Fig: mRNA level of *NANOG* and *OCT4* before and after transfection.
